# Article-processing charges as a barrier for science in low-to-medium income regions

**DOI:** 10.1590/0074-02760220064

**Published:** 2022-06-17

**Authors:** Marcio L Rodrigues, Wilson Savino, Samuel Goldenberg

**Affiliations:** 1 Fundação Oswaldo Cruz-Fiocruz, Instituto Carlos Chagas, Curitiba, PR, Brasil; 2 Universidade Federal do Rio de Janeiro, Instituto de Microbiologia Paulo de Góes, Rio de Janeiro, RJ, Brasil; 3 Fundação Oswaldo Cruz-Fiocruz, Instituto Oswaldo Cruz, Laboratório de Pesquisas sobre o Timo, Rio de Janeiro, RJ, Brasil; 4 Fundação Oswaldo Cruz-Fiocruz, Instituto Oswaldo Cruz/Instituto Nacional de Ciência e Tecnologia em Neuroimunomodulação, Rio de Janeiro, RJ, Brasil; 5 Fundação Oswaldo Cruz-Fiocruz, Instituto Oswaldo Cruz, Rede FAPERJ de Pesquisa em Neuroinflamação, Rio de Janeiro, RJ, Brasil

**Keywords:** author processing charges, open access, low-to-medium income countries

## Abstract

It is widely accepted that science is universal by nature. However, to make science universal, access to research findings is imperative. The open access model of publication of academic articles was established and consolidated during the last two decades. However, most of the open access journals apply article-processing charges (APCs), which can cost more than USD 10,000.00. In regions where support for research is scarce, these funds are usually not available. Similar problems occur in countries with weak economies and, consequently, unfavorable currency conversion rates. This situation reveals a barrier to the alleged universality of science and the access to research findings. In this manuscript, the barriers faced by authors and institutions from low-to-middle income regions to cover APCs and make their science freely available are discussed and illustrated with recent numbers.

Article processing charges (APCs) correspond to a fee paid by the authors of academic articles, their research funders, or institutions during the publication process.^1^ APCs are directly connected to the private open-access model of publication, where an article that is considered suitable for publication is made available online and free to read for anyone with an internet connection. Usually, the article is made available under a Creative Commons CC-BY license, a tool that allows for anyone to reuse, share, or build upon the work.^2^ APCs are used by private open access journals to pass up the subscription costs that libraries and readers habitually had paid to have access to academic articles. Therefore, APCs allowed the transfer of journal production costs (article production, editorial management, peer review systems, dissemination of papers on online platforms or journal websites) from readers to authors.^1^ Charging APCs allows publishers of different nature (academic, corporate, non-profit, and scientific societies, among others) to meet their income needs and publishing costs. This model differs from the traditional system of page charges, which were (and are still) used to cover administrative costs in addition to the cost of print publication. However, page charges do not make the articles freely available as they are in the open access/APCs model.^1^

**Publish or perish - and pay**

The process of publication^3^ and its connection with APCs can be simplified as follows. Authors of scholarly studies first fight to obtain research funding from public and/or private sources to develop their research, then they make their results available for voluntary editorial management and peer review by the scientific journals. When these results are considered scientifically sound and allegedly arise from procedures of high ethical standards, they can be published by the scientific journal where they were initially submitted. In the open-access model, the authors (or their institutions or funders) pay APCs to make these results accessible to any reader. The journals, on the other edge, normally conduct the whole editorial process of peer review based on highly qualified - but voluntary - work.^4^ It is important noting that several if not most editorial companies that charge for APCs have a low manuscript editing cost in view of engaged staff, and printing costs. The reviewers, probably the most important players in this process of manuscript submission, receive no financial retribution for their work. This last point is, in fact, a paradox since despite their work as reviewers, scientists are in general fully charged when they decide to publish in that given journal.

Not all journals are open access and consequently do not charge APCs. Therefore, the reasons by which authors decide to pay APCs are multiple.^1^ For instance, open access can increase the readership of the article, and increased access can lead to higher citation rates, a well-known index of success in the scientific career.^5^ In addition, the more a given paper is read by different sectors, the more likely it will be useful for the benefit of the people. Furthermore, paying APCs is commonly a demand from funding agencies. Several funders in Europe in the US require open access publication as a condition to connect public funding to the dissemination of science. Overall, APCs represent a major paradox in the dissemination of science, since the authors, their institutions, or funders must pay to make their research freely available.

The discussion on the currently practiced open access models necessarily includes the so-called Plan S, a set of requirements drafted in 2018 by 11 national funding agencies across Europe collectively called cOAlition S.^6^ The group’s aims were virtuous: their initiative was dedicated to making scientific research publicly available, ending the reign of paywalls, and promoting a transition to a fully open-access publishing model in science. Plan S mandates that newly published studies are made open access without a waiting period, and that funders must cover grantees’ APCs.^7^

There are important concerns on the consequences of Plan S implementation. For instance, it was proposed that Plan S is simply promoting a move from a reader-pays to an author-pays system.^7^ Other outcomes of the Plan S mandate are also a reason of concern.^8^ As well portraited by Alejandra Manjarrez, APCs among journals with higher impact factors faced an explosion in the last decade.^7^ At journals in the upper 50% of Scopus classification, APCs increased more than 80%. Journals’ APCs similarly ranked in the Journal Citation Reports increased more than 130%.^9^ This information is compatible with the findings by Khoo, who demonstrated that ACPs paid by European institutions between 2005 and 2018 grew significantly higher than the 2005 fee indexed according to inflation in the United States and Europe.^10^

**How much does it cost to make science available?**

In a recent essay, the problems faced by authors from low-to-medium income regions to publish open access articles were proficiently discussed.^11^ It is clear for us, authors included in such a situation, that covering the costs for publishing open access is indeed a major barrier to making our science accessible. This is an issue faced by scientists from dozens of low-to-medium income countries. For instance, APCs are purportedly a major obstacle for the progress of African science.^12^ Among several others, the Brazilian model of research funding efficiently illustrates - with numbers - how hard is to cover the costs of equipment and consumables and still pay for APCs. A discussion of this model and the difficulties faced by authors working under these conditions follows below. This rationale is mostly based on Brazil, but it reflects the difficulties of several other countries under similar conditions.

Under the currently practiced models, it is important to highlight that the fee structure of Plan S is unrealistic in countries like Brazil. As well discussed by Kowaltowski and Oliveira, APCs in Brazil are not supported by supplementary funds, but by subtraction from grant totals.^13^ That means that the authors must opt between consumables and APCs, which is undeniably prejudicial for science. Plan S includes “to establishing fair and reasonable prices for publishing services, including equitable waiver policies, that reflect the publishing costs”,^6^ but the regular practices of paying APCs reveals that completely variable criteria are used by different publishers to grant waivers for authors from low-to-middle income countries. In fact, plan S also states that upper-middle-income countries will be excluded from the waiver policies,^6^ which likely consists of a rigid and outdated classification that will negatively impact authors from those countries. Remarkably, the problem of Brazilian scientists being denied in their requests for waivers and discounts efficiently illustrates this situation.^14^ Therefore, authors from countries deemed “too rich” for fee waivers will have subscription journals as the only publishing option, as recently discussed.^15^

The National Council for Scientific and Technological Development (CNPq) is a public foundation linked to the Ministry of Science, Technology, and Innovation to support Brazilian research. CNPq launched in 2021 the “Universal” call, its most traditional program for funding research in all areas.^16^ This call received 8,877 proposals and approximately 15% of them were supported. It was divided into proposals from senior and emerging groups. Proposals from senior groups were limited to a maximum of approximately USD 50,000 (today’s values used for the conversion from Brazilian Reais to US dollars) for 36 months. To ask for USD 50,000, the team had to necessarily include 9 researchers holding a Ph. degree. That gives total financial support of less than USD 6,000 per investigator in the team, or something close to USD 2,000 per scientist each year. Particularly in experimental sciences, this funding is very, very far below the minimum.

A median cost of APCs equal to USD 2,600 was recently estimated.^11^ However, APCs can cost more than USD 10,000.^17^ It is noteworthy mentioning that the funding models discussed herein for the Brazilian system and others are not supposed to cover only APCs, but everything else. In summary, it is virtually impossible to cover APCs under these conditions, although it means exclusion from publishing open access papers. The problem is not new. Six years ago, we raised this discussion when 1 USD corresponded to 3.5 Brazilian Reais.^18^ After a huge financial crisis and controversial governmental decisions that are directly affecting Brazilian science,^19^ today’s conversion rate indicates that 1 USD corresponds to more than 5 Brazilian Reais, but the prices remain the same in the northern hemisphere currencies (American Dollars, Euros, Swiss francs, British Pounds, etc). Of note, currency conversion is only part of the problem since the values of APCs have been constantly increasing in high-reputation journals over the years.^7^ ^,^ ^9^ ^,^ ^10^ It was recently demonstrated that is economically viable for major publishers to waive APCs for authors from low-to-medium income regions;^20^ now it is time for concrete actions.

Suggestions on how to mitigate the problem are hard to elaborate. Above all, publishing in journals associated to scientific societies has been consistently proposed as an important action,^13^ since these publishers are usually non-profit and take science itself above publication costs. The authors, in fact, play an important role in this process. As stated in Plan S, and in accordance with the San Francisco Declaration on Research Assessment (DORA), it is fundamental to value “the intrinsic merit of the work and not consider the publication channel, its impact factor (or other journal metrics), or the publisher”.^6^ According to this principle, where to publish is less important than what to publish. To make this principle realistic, the authors are the most important players at the initial stage of the publication process: avoiding journals that charge inexplicably high APCs would be mandatory, independently on impact factors or similar metrics. However, funding agencies and the academy in general play fundamental roles in this process too, since they tend to give more appreciation to journal records than the findings described there. A major change in the evaluation process is required to stimulate authors to choose where to publish independently on the common sense imposed by the high impact journals.^21^ Of note, evaluation committees (both for grants and hiring scientists) are composed by scientists, who are ultimately authors.

**In conclusion**

To be inclusive, the APC system must change, and this is urgent. If not, scientists from low-to-medium income regions will not have their science known by the scientific community and the people in general. This situation strikingly contrasts with the widely accepted concept that science is universal by nature,^22^ and enhances the already existence of inequalities in science, negatively impacting the scientific-derived benefits for the people(s).
